# Serum NLRP3 levels are associated with postoperative delirium of elderly patients with lung cancer: an observational analytical study

**DOI:** 10.3389/fneur.2026.1868771

**Published:** 2026-09-29

**Authors:** Yuanchao Cheng, Yuzhen Liu, Guanglin Tan, Mingjiang Huang, Xuhui Wu, Xiaofeng Chen

**Affiliations:** 1 Department of Cardiothoracic Surgery, The First Affiliated Hospital of Lishui University, Lishui People’s Hospital, Lishui, Zhejiang, China; 2 Department of Intensive Care Unit, Longquan People’s Hospital, Lishui, Zhejiang, China; 3 Department of Surgery, Suichang County Hospital of Traditional Chinese Medicine, Lishui, Zhejiang, China

**Keywords:** elderly, lung cancer, NOD-like receptor pyrin domain-related protein 3, postoperative delirium, serum

## Abstract

**Objective:**

NOD-like receptor protein 3 (NLRP3) regulates the inflammatory response. This study aimed to investigate the relationship between perioperative serum NLRP3 levels and postoperative delirium (POD) in elderly patients with lung cancer.

**Methods:**

In this observational analytical study, serum NLRP3 levels were measured preoperatively and postoperatively in 266 elderly patients with lung cancer and at study entry in 100 controls. We used the American Society of Anesthesiologists (ASA) Scale, Charlson Comorbidity Index (CCI), and Eastern Cooperative Oncology Group (ECOG) Scaleto classify patients’ preoperative physical fitness. Patients were assessed for POD from the first to the third postoperative day using the Chinese Confusion Assessment Method. Predictors of POD were determined using multivariate analysis.

**Results:**

Preoperative and postoperative serum NLRP3 levels in patients were significantly higher than those in controls, with the highest levels observed postoperatively. Postoperative blood C-reactive protein levels and ECOG scores were independently correlated with postoperative serum NLRP3 levels. Postoperative serum NLRP3 levels, ASA classifications, and CCI were independently associated with POD. The nomogram model incorporating the three independent predictors of POD was confirmed to have good discrimination, calibration, and clinical utility based on the receiver operating characteristic curve, calibration curve, and decision curve. Compared with the clinical model (CCI + ASA), the nomogram model demonstrated significant improvement in risk reclassification, with a continuous NRI of 0.7198 (95% CI, 0.4366–1.003) and an IDI of 0.0867 (95% CI, 0.041–0.1323); however, the improvement in AUC did not reach statistical significance. Internal validation through 10-fold cross-validation demonstrated that the nomogram model had good predictive accuracy based on the optimism-corrected area under the curve.

**Conclusion:**

Significantly increased postoperative serum NLRP3 levels are independently associated with POD in elderly patients with lung cancer, and the model integrating serum NLRP3 levels shows favorable predictive performance for POD.

## 1 Introduction

Lung cancer, a common malignancy, poses a substantial burden on health, society, and the economy worldwide (1, 2). Postoperative delirium (POD) is often encountered in elderly patients, especially in those with cancer (3). POD can apparently prolong the length of hospitalization, increase medical and caregiving expenses, and even increase the risk of a poor prognosis in cancer patients (4). Multiple factors contribute to POD, among which functional metrics with a health sense are important determinants of POD because poor health status obviously increases vulnerability to injury (5). For cancer patients, the Eastern Cooperative Oncology Group (ECOG) scale may be a clinically useful measure of functional status. The pathophysiological mechanisms of POD are rather complex, while inflammatory insults, possibly leading to imperceptible brain injury, have been corroborated as an important factor in the pathogenesis of POD (6–8). Besides therapeutic interventions, early and accurate prediction of POD is an imperative step during the aggressive management of cancers (5). As such, blood biomarkers have received extensive attention regarding their predictive ability for POD, given the convenient availability of blood samples (9).

The NOD-like receptor protein 3 (NLRP3) inflammasome, a central regulator of innate immune and inflammatory signaling, participates in the pathophysiological processes of several inflammation-related illnesses, such as sepsis, acute myocardial infarction, and cancers (10). NLRP3 inflammasome signaling has been implicated in lung cancer biology through its regulation of inflammatory signaling and the tumor immune microenvironment (11). Specifically, greater activation of the NLRP3-associated peritumoral inflammatory pathway has been correlated with shorter overall survival in early-stage non-small cell lung cancer, and NLRP3-dependent inflammation may facilitate lung tumor development and growth (12–14). In addition, elevated NLRP3 levels were highly associated with a poor prognosis in patients with lung cancer (15). Also, elevated serum NLRP3 levels were firmly associated with increased disease severity and worse neurological outcomes in cerebral ischemia, spontaneous intracerebral hemorrhage, aneurysmal subarachnoid hemorrhage, and traumatic brain injury (16, 17). Furthermore, disturbances in peripheral immunity are intimately relevant to brain dysfunction; and peripheral NLRP3 inflammasome activation has been affirmed as a causal pathway in the development of cognitive disorders because it produces pro-inflammatory cytokines like interleukin-1beta, and its activation confers adverse effects not only on the periphery but also on the brain (18, 19). Collectively, serum NLRP3 is likely to act as a biomarker of brain injury, thereby potentially predicting POD. In this study, serum NLRP3 levels were measured in elderly patients with lung cancer to explore their predictive value for POD.

## 2 Materials and methods

### 2.1 Study design, participant selection, and ethical considerations

This observational analytical study was conducted at the First Affiliated Hospital of Lishui University between January 2025 and December 2025. Elderly patients with lung cancer and elderly controls were enrolled, and the study was divided into a prospective cohort sub-study and a cross-sectional sub-study to determine the clinical significance of serum NLRP3 in lung cancer. Inclusion criteria for patients were (1) age of at least 65 years, (2) newly diagnosed primary lung cancer, and (3) the need for elective thoracoscopic surgery. Exclusion criteria for patients were (1) previous neuropsychiatric diseases, such as dementia, psychosis, delirium, Parkinson’s disease, intracranial infection, stroke, traumatic brain injury, intracranial space-occupying lesions, and others; (2) systemic diseases or severe diseases of other organs, for example, malignancies, heart failure, liver cirrhosis, uremia, ascites, and so forth; (3) other specific conditions, for instance, alcohol or drug dependence, trauma, surgery, or infection within the recent 1 month, use of hormones or immunoinhibitory drugs, unavailable blood samples, incomplete clinical data, refusal to participate, and loss to follow-up. Controls were recruited according to the following eligibility criteria: (1) age of 65 years or greater; (2) absence of severe illnesses, e.g., malignancies, heart failure, liver cirrhosis, uremia, ascites, and others; however, common chronic comorbidities such as hyperlipidemia, hypertension, and diabetes mellitus, were not excluded; (3) no obvious abnormalities in conventional tests, such as electrocardiogram, chest radiograph, blood leucocyte counts, blood hemoglobin levels, and other routine tests. The study was implemented in accordance with the principles of the Declaration of Helsinki and its later updates. The Ethics Committee of the First Affiliated Hospital of Lishui University reviewed and approved the study protocol (Approval Number, 2025–061-01). Participants were fully informed about the study objectives and procedures and voluntarily provided written informed consent.

### 2.2 Clinical data collection and POD identification

The collected baseline data included age, sex, height, weight, smoking status, alcohol consumption, and histories of hypertension, diabetes mellitus, and hyperlipidemia. The American Society of Anesthesiologists (ASA) Physical Status Classification, Charlson comorbidity index (CCI), and Eastern Cooperative Oncology Group (ECOG) performance status were adopted to classify patients’ preoperative physical status (20–22). ECOG performance status and ASA physical status were clinician-rated according to their standard definitions, and the CCI was calculated from documented comorbidities using the established weighting system. As applicable, patients underwent wedge resection, segmentectomy, lobectomy, or pneumonectomy. In addition, operative duration, intraoperative blood loss, and total propofol consumption were documented. The confusion assessment method (CAM) has demonstrated acceptable reliability and diagnostic validity for delirium (23). Using CAM, patients were assessed twice daily (9:00 a.m. and 4:00 p.m.) during the first three postoperative days by a physician trained in CAM administration who was blinded to the serum NLRP3 results; any positive CAM assessment within postoperative days 1–3 was defined as POD (24, 25). In this study, the primary outcome was POD, and the secondary outcome was serum NLRP3 levels.

### 2.3 Immunoassay

Fasting blood samples were collected from patients in the early morning of preoperative day 1 and postoperative day 1 before CAM assessment, and from controls at study entry. Some conventional parameters, including blood leucocyte counts, blood C-reactive protein (CRP) levels, and blood glucose levels, were measured using routine laboratory methods. To quantify serum NLRP3 levels, blood samples, following natural clotting, were separated by centrifugation at 3000 × g for 10 min, and the serum specimens were subsequently stored at −80 °C until analysis. All samples were thawed within 3 months of collection. Thereafter, serum NLRP3 levels were measured using an enzyme-linked immunosorbent assay by the same professional technician, who were blinded to the study data. The kit was purchased from CUSABIO TECHNOLOGY LLC., Houston, USA (Catalogue number, CSB-E15885H), with a detection range of 0.156–10.0 ng/mL and sensitivity of 0.039 ng/mL. In this study, the spike-recovery rate was 96.21%; the coefficient of determination (R^2^) for dilution linearity was 0.97; the intra-assay coefficient of variation (CV) was 5.85%; and inter-assay CV was 7.94%. Each sample was measured in duplicate on the same plate, and the mean of the two measurements was used for statistical analysis. The CV between duplicate measurements was calculated as the standard deviation of the duplicate values divided by their mean and multiplied by 100%. Duplicate measurements were accepted when the CV was ≤10%. All samples were measured in duplicate. In the initial measurements, the duplicate CVs ranged from 3.27 to 13.63% (mean, 6.45%; standard deviation, 2.06%). According to the predefined quality-control criterion, samples with a duplicate CV > 10% were re-assayed. A total of eight samples required re-assay. After re-assay, the duplicate CVs ranged from 3.27 to 9.53% (mean, 6.22%; standard deviation, 1.72%), and all re-assayed samples met the predefined CV criterion of ≤10%.

### 2.4 Sample size calculation

Based on the previous report and preliminary clinical observations, the incidence of POD among elderly patients undergoing lung cancer surgery was approximately 20% (26). In accordance with the criterion of 10 events per variable and three predictors of POD, approximately 30 POD events and at least 150 patients were considered necessary. We considered sample size before model development, independently of the number of predictors ultimately retained in the final model. Following completion of enrollment and data collection, candidate variables associated with POD in the univariable analyses were subjected to LASSO regression with 10-fold cross-validation for data-driven variable selection. Thus, the three predictors included in the final POD model were identified after enrollment and were not used to determine the prespecified sample size. Separately, an *a priori* power analysis was performed using G*Power version 3.1.9.4 for to compare serum NLRP3 levels between independent groups. Because sufficiently comparable prior data were unavailable, a standardized effect size of 0.80 was assumed according to Cohen’s conventional definition of a large effect size. With a two-sided α of 0.05 and statistical power of 95%, at least 72 participants per group were required. The final study included 266 patients and 100 controls, exceeding these prespecified minimum sample-size targets.

### 2.5 Statistical analysis

Statistical analyses were performed using SPSS version 25.0 for conventional univariate and multifactorial analyses, GraphPad Prism version 9.0 for graphing, MedCalc version 17.4 for receiver operating characteristic curve (ROC) analysis, and R version 4.6.1 (packages: ggrcs, rms, hmisc and others) for restricted cubic spline analysis, nomogram construction, calibration curve analysis, decision curve analysis, and other analyses. All numerical variables were non-normally distributed according to the Kolmogorov–Smirnov test and, accordingly, were expressed as medians (lower–upper quartiles); they were compared using the Mann–Whitney U-test between two independent groups, and the Kruskal–Wallis test among multiple groups. The Wilcoxon signed-rank test was used to compare paired preoperative and postoperative measurements in the same patients. Categorical variables are presented as numbers (percentages) and were compared between two groups using the chi-square test or Fisher’s exact test, as appropriate. Spearman’s rank correlation test was used to assess bivariate correlations.

Serum NLRP3 levels underwent logarithmic transformation. With serum NLRP3 levels and POD as dependent variables, those factors that were significantly correlated with or differed in univariate analyses, were identified. And then, variable selection was then further performed using the least absolute shrinkage and selection operator (LASSO) regression model. Ten-fold cross-validation was conducted to identify the optimal penalty parameter (lambda). Based on the lambda value within one standard error of the minimum, predictors with non-zero coefficients were considered relevant and included in the multifactorial linear regression and binary logistic regression models, respectively, to ascertain factors that were independently related to serum NLRP3 levels and POD.

Restricted cubic spline analysis using four knots was conducted for evaluating the linear relationships between serum NLRP3 levels and other variables. The variance inflation factor (VIF) was computed to assess multicollinearity in the regression analysis. The Brier score was calculated to assess the goodness of fit of the regression model. The E-value was computed to determine the strength of unmeasured confounding necessary to negate the observed results. Discrimination performance was evaluated based on ROC curve analysis, and the optimal cutoff values of various metrics were generated via the Youden index.

The nomogram model of POD incorporating its independent predictors had its calibration correctness assessed by calibration curve analysis, its discrimination performance assessed by ROC curve analysis, and its clinical utility assessed by decision curve analysis. In addition to the nomogram model, another model incorporating only the integrating clinical variables from multivariate analysis was constructed. The area under the ROC curve was calculated to assess the performance of the two models and was compared by DeLong’s test. Continuous (category-free) net reclassification improvement (NRI) and integrated discrimination improvement (IDI) were used to quantify the incremental predictive value of the nomogram model compared with the clinical model. The 95% confidence intervals for NRI and IDI were estimated using bootstrap resampling with 1,000 replicates. Because continuous NRI represents the sum of the reclassification improvements among participants with and without POD, its theoretical range is −2 to 2; therefore, its confidence interval may extend beyond 1. Moreover, 10-fold cross-validation performed to obtain an unbiased estimate of the model’s prediction accuracy, and the optimism-corrected area under the curve was reported. Furthermore, the goodness-of-fit of models was qualitatively determined through Akaike information criterion (AIC) and Bayesian information criterion (BIC).

The prospective cohort study was characterized by a low likelihood of missing values, and therefore the complete-case method was adopted for handling missing values. For all analyses, two-sided *p*-values < 0.05 were deemed as the presence of statistically significant differences.

## 3 Results

### 3.1 Study subjects and basic characteristics

During the study period, 348 elderly patients with newly diagnosed primary lung cancer who were scheduled for elective thoracoscopic surgery met the initial inclusion criteria. Of these, 82 were excluded due to reasons delineated in Figure 1. Consequently, 266 patients were included in the final cohort analysis. POD was assessed during postoperative days 1–3. Of the 266 patients, 53 developed POD and 213 remained free of POD. Table 1 shows baseline characteristics of patients. A total of 100 controls had median age of 71 (lower-upper quartiles, 67–73 years), including 54 males and 46 females, had median body mass index of 24.4 kg/m^2^ (lower-upper quartiles, 23.0–25.8 kg/m^2^), had median blood leucocyte counts of 5.8 × 10^9^/L (lower-upper quartiles, 4.9–6.9 × 10^9^/L), had median blood CRP levels 3.3 mg/L (lower-upper quartiles, 2.0–6.0 mg/L), had median blood glucose levels 5.9 mmol/L (lower-upper quartiles, 5.2–6.7 mmol/L), had median serum NLRP3 levels 0.52 ng/mL (lower-upper quartiles, 0.42–0.70 ng/mL), and included 21 tobacco smokers (21.0%), 26 alcohol drinkers (26.0%), 34 hypertensive cases (34.0%), 12 diabetic individuals (12.0%), and 24 hyperlipidemic subjects (24.0%). No significant differences were observed between patients and controls for the above factors (all *p* > 0.05).

**Figure 1:**
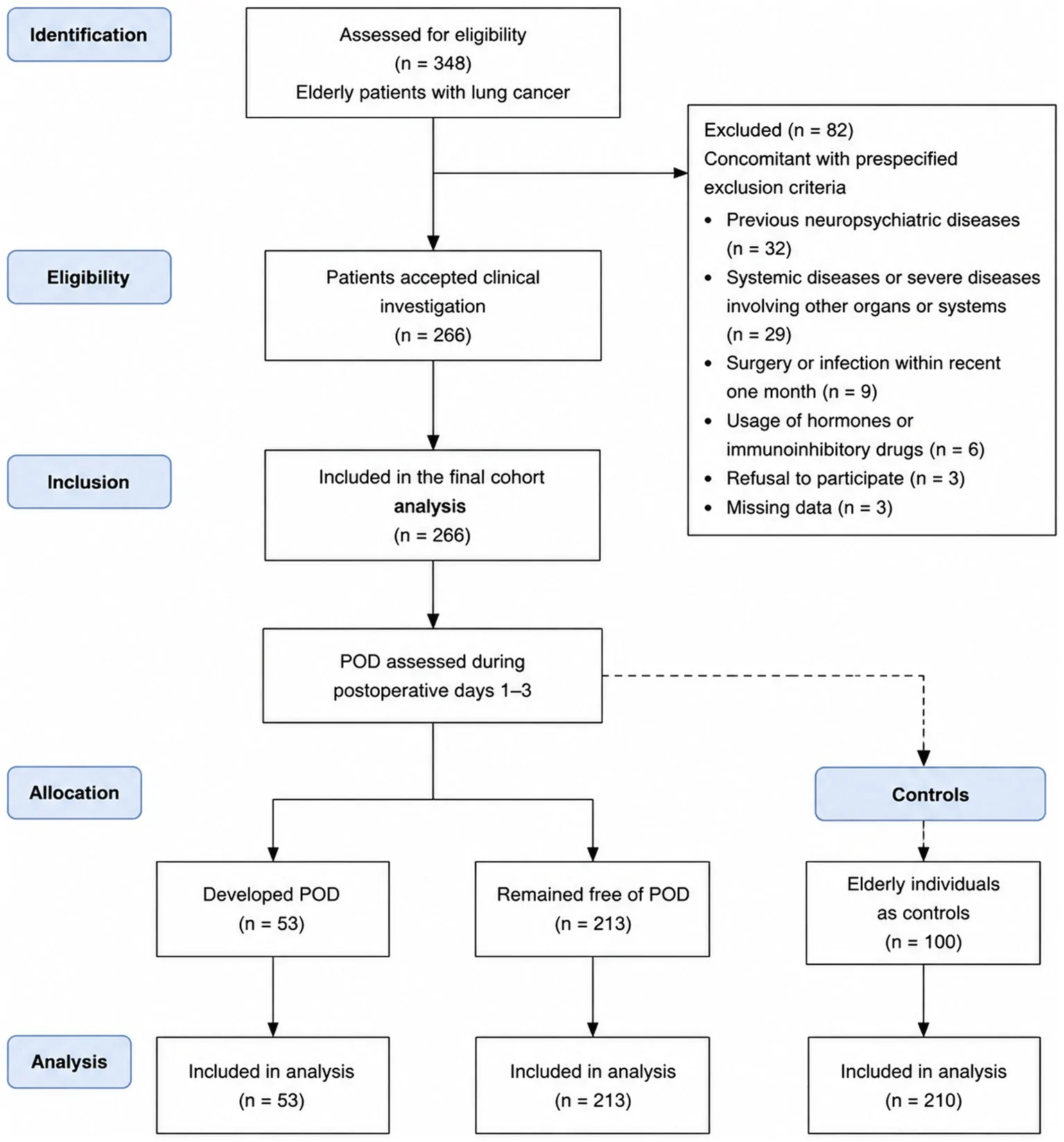
Flowchart of selecting eligible elderly patients with lung cancer. Initially, a total of 348 patients were assessed, and afterward, 82 patients were excluded. Ultimately, 266 patients were enrolled for further analysis. NLRP3 indicates NOD-like receptor protein 3.

**Table 1:** Baseline characteristics of patients and bivariate correlation analysis of postoperative serum NLRP3 levels.

Parameters	All patients	Correlation with serum NLRP3 levels	
		ρ	*p* value
Age (years)	71 (68–74)	0.092	0.136
Gender (male/female)	148/118	0.067	0.279
Body mass index (kg/m^2^)	23.6 (21.3–25.8)	−0.068	0.273
Tobacco smoking	60 (22.6%)	0.055	0.375
Alcohol drinking	72 (27.1%)	−0.011	0.854
Hypertension	104 (39.1%)	0.090	0.144
Diabetes mellitus	42 (15.8%)	0.063	0.304
Hyperlipidemia	76 (28.6%)	−0.001	0.981
Lung resection method (wedge resection/segmentectomy/lobectomy/pneumonectomy)	95/97/67/7	0.099	0.107
Surgical time (min)	98 (73–128)	0.091	0.140
Intraoperative blood loss (mL)	80 (64–125)	0.112	0.068
Propofol dosage (mg)	797 (669–1,053)	0.108	0.079
Arterial systolic pressure (mmHg)	133 (128–139)	−0.028	0.653
Arterial diastolic pressure (mmHg)	79 (74–82)	−0.105	0.089
American society of anesthesiologists classifications	2 (1–2)	0.318	<0.001
Eastern cooperative oncology group scores	1 (1–2)	0.526	<0.001
Charlson comorbidity index scores	3 (2–4)	0.239	<0.001
Preoperative blood leucocyte counts (×10^9^/L)	7.1 (6.2–8.0)	0.105	0.086
Preoperative blood C-reation protein levels (mg/L)	5.7 (3.9–10.0)	0.214	<0.001
Preoperative blood glucose levels (mmol/L)	5.3 (4.4–6.2)	0.091	0.139
Preoperative serum NLRP3 levels (ng/mL)	1.6 (1.3–2.2)	0.252	<0.001
Postoperative blood leucocyte count (×10^9^/L)	8.7 (7.6–9.9)	0.090	0.144
Postoperative blood C-reation protein levels (mg/L)	8.4 (6.0–14.5)	0.509	<0.001
Postoperative blood glucose levels (mmol/L)	5.9 (4.2–7.3)	0.118	0.054

Variables were presented as counts (percentages) or medians (percentiles 25th-75th) as applicable. Correlations were appraised using Spearman test. NLRP3 indicates NOD-like receptor protein 3.

### 3.2 Variation of serum NLRP3 levels and their correlates in lung cancer patients

As shown in Figure 2, patients’ preoperative serum NLRP3 levels (median, 1.63 ng/mL; lower- upper quartiles, 1.28–2.16 ng/mL) and postoperative serum NLRP3 levels (median, 4.25 ng/mL; lower-upper quartiles, 3.59–5.30 ng/mL) were significantly higher than those of controls (median, 0.52 ng/mL; lower-upper quartiles, 0.42–0.70 ng/mL) (all *p* < 0.001); and moreover, postoperative, in contrast to preoperative, serum NLRP3 levels were substantially higher than preoperative levels (*p* < 0.001). Additionally, postoperative serum NLRP3 levels were markedly positively correlated with ECOG scores (ρ = 0.526, *p* < 0.001; Supplementary Figure 1). In accordance with the ECOG scale, patients were subdivided into four subgroups. The higher the ECOG scores, the higher the postoperative serum NLRP3 levels (*p* < 0.001; Supplementary Figure 2). Alternatively, postoperative serum NLRP3 levels were notably positively correlated with postoperative blood CRP levels (ρ = 0.509, *p* < 0.001; Supplementary Figure 3). In the restricted cubic spline analysis, postoperative serum NLRP3 levels were linearly related to ECOG scores (p for non-linear = 0.225; Supplementary Figure 4) and postoperative blood CRP levels (p for non-linear = 0.072; Supplementary Figure 5). Table 1 shows that postoperative serum NLRP3 levels were also highly correlated with ASA classifications (ρ = 0.318, *p* < 0.001), CCI scores (ρ = 0.239, *p* < 0.001), preoperative blood CRP levels (ρ = 0.214, *p* < 0.001), and preoperative serum NLRP3 levels (ρ = 0.254, *p* < 0.001). As shown in Supplementary Figures 6, 7, the preceding six variables were entered into the LASSO model, and ECOG scores and postoperative serum CRP levels were selected as the final variables for inclusion in the multivariable linear regression model. As a result, postoperative serum NLRP3 levels were independently correlated with ECOG scores (beta, 0.071; 95% confidence interval (CI), 0.050–0.093; *p* < 0.001; VIF = 1.158) and postoperative blood CRP levels (beta, 0.009; 95% CI, 0.006–0.013; *p* < 0.001; VIF = 1.158).

**Figure 2:**
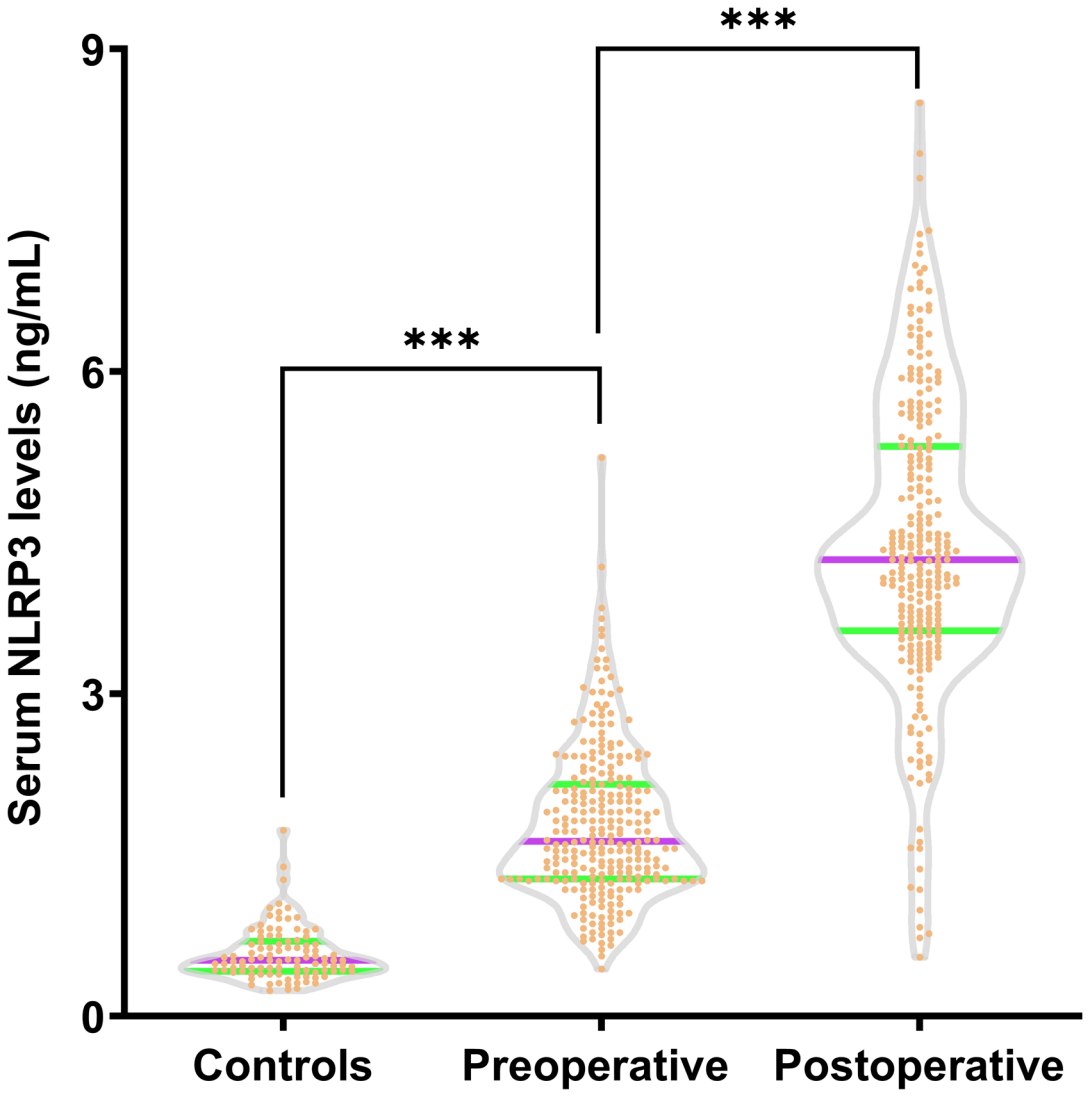
Box plot shows serum NLRP3 levels among different groups. Elderly patients with lung cancer, relative to controls, had notably increased postoperative and preoperative serum NLRP3 levels, with postoperative levels being significantly highest (all ^***^p < 0.001). NLRP3 refers to NOD-like receptor protein 3.

### 3.3 Association between postoperative serum NLRP3 levels and POD

In total, 53 patients development POD. As outlined in Supplementary Figure 8, patients with POD had significantly higher postoperative serum NLRP3 levels than those without POD (median, 5.60 ng/mL vs. 4.10 ng/mL; *p* < 0.001). Additionally, postoperative serum NLRP3 levels >5.1 ng/mL predicted POD with a Youden index of 0.520 (Figure 3). Moreover, postoperative serum NLRP3 levels were associated with the likelihood of POD in a dose-effect relationship (p for non-linear = 0.087; Figure 4). As shown in Table 2, postoperative serum NLRP3 levels, propofol dosage, ASA classifications, CCI, ECOG scores, preoperative serum CRP levels, preoperative serum NLRP3 levels, and postoperative serum CRP levels differed significantly between patients with POD and those without the complication (all *p* < 0.05). As shown in Supplementary Figures 9, 10, the above eight variables were entered into the LASSO model, and ASA classifications, CCI and postoperative serum NLRP3 levels were chosen as final variables for entry into the binary logistic regression model. As a consequence, postoperative serum NLRP3 levels (odds ratio, 1.879; 95% CI, 1.400–2.523; *p* < 0.001; VIF = 1.126), ASA classifications (odds ratio, 2.317; 95% CI, 1.313–4.089; *p* = 0.004; VIF = 1.339) and CCI (odds ratio, 1.877; 95% CI, 1.297–2.717; *p* = 0.001; VIF = 1.293) were independently predictive of POD. Brier score was 0.1053. In Supplementary Figure 11, the E value was equal to 3.1642 (95% CI, 2.1483–4.4832). As shown in Supplementary Figure 12 and Table 3, postoperative NLRP3 levels possessed a POD-prediction ability comparable to those of ASA classification and CCI (both *p* > 0.05). The model integrating the three independent predictors of POD was represented by the nomogram (Figure 5). Calibration curve analysis demonstrated acceptable calibration (Figure 6), and decision curve analysis showed good clinical utility (Figure 7). As shown in Supplementary Figure 12 and Table 3, the predictive capability of the nomogram model significantly exceeded that of the above three metrics alone (all *p* < 0.05), but did not exceed that of the model combining CCI with ASA classifications (*p* > 0.05). In Supplementary Figure 13, NRI was 0.7198 (95% CI, 0.4366–1.003; *p* < 0.001) and IDI was 0.0867 (95% CI, 0.041–0.1323; *p* < 0.001). In Supplementary Figure 14, 10-fold cross-validation confirmed that the optimism-corrected area under the curve was 0.825 (95% CI, 0.752–0.899). The nomogram model (AIC = 195.16, BIC = 209.49) performed better than the model incorporating only CCI and ASA (AIC = 212.93, BIC = 223.68).

**Figure 3:**
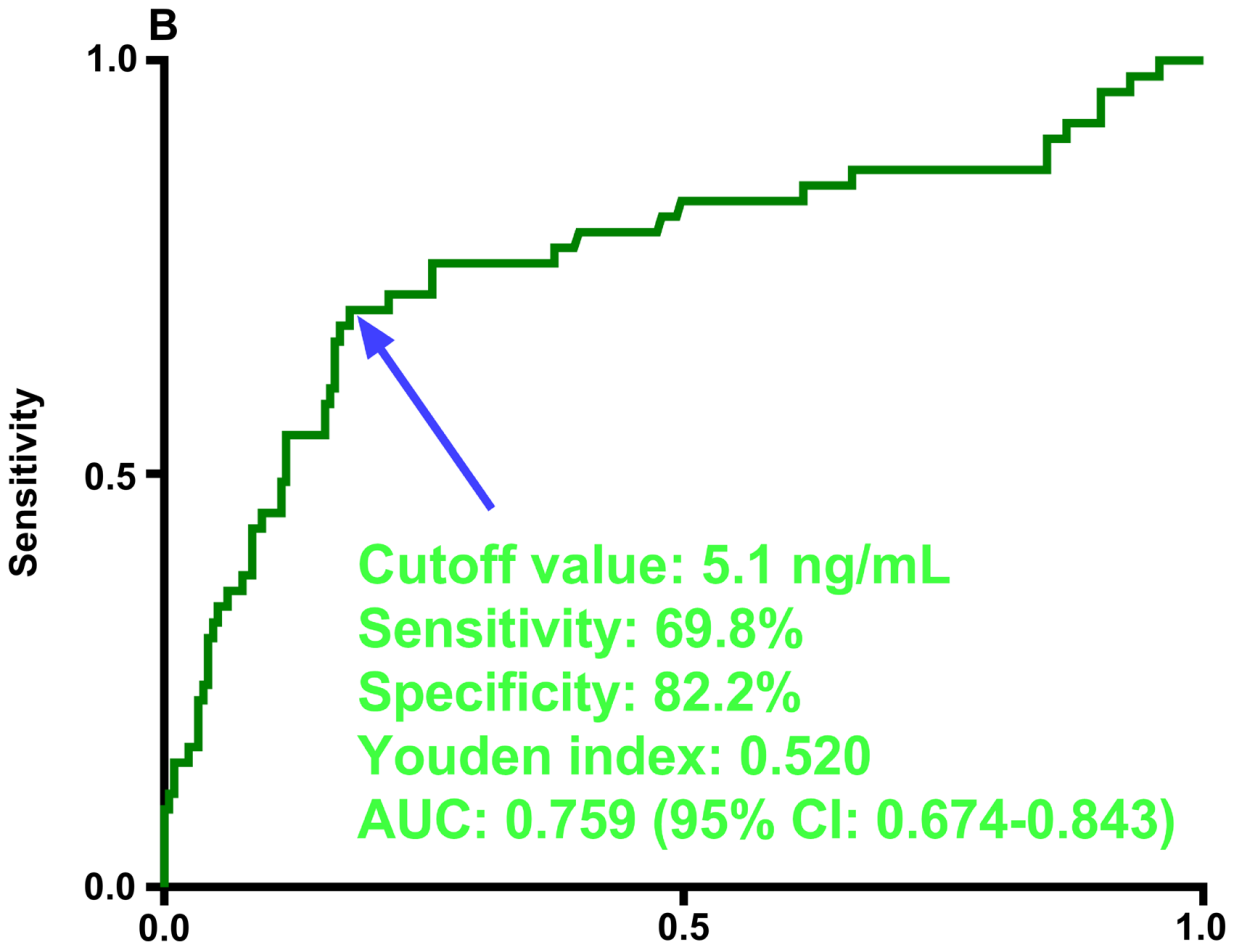
Receiver operating characteristic curve shows the predictive value of postoperative serum NLRP3 levels for postoperative delirium. Postoperative delirium was efficiently predicted using postoperative serum NLRP3 levels, and an appropriate criterion was identified using the Youden method. AUC means area under the curve; 95% CI, 95% confidence interval; NLRP3, NOD-like receptor protein 3. Blue arrow points to the cutoff value.

**Figure 4:**
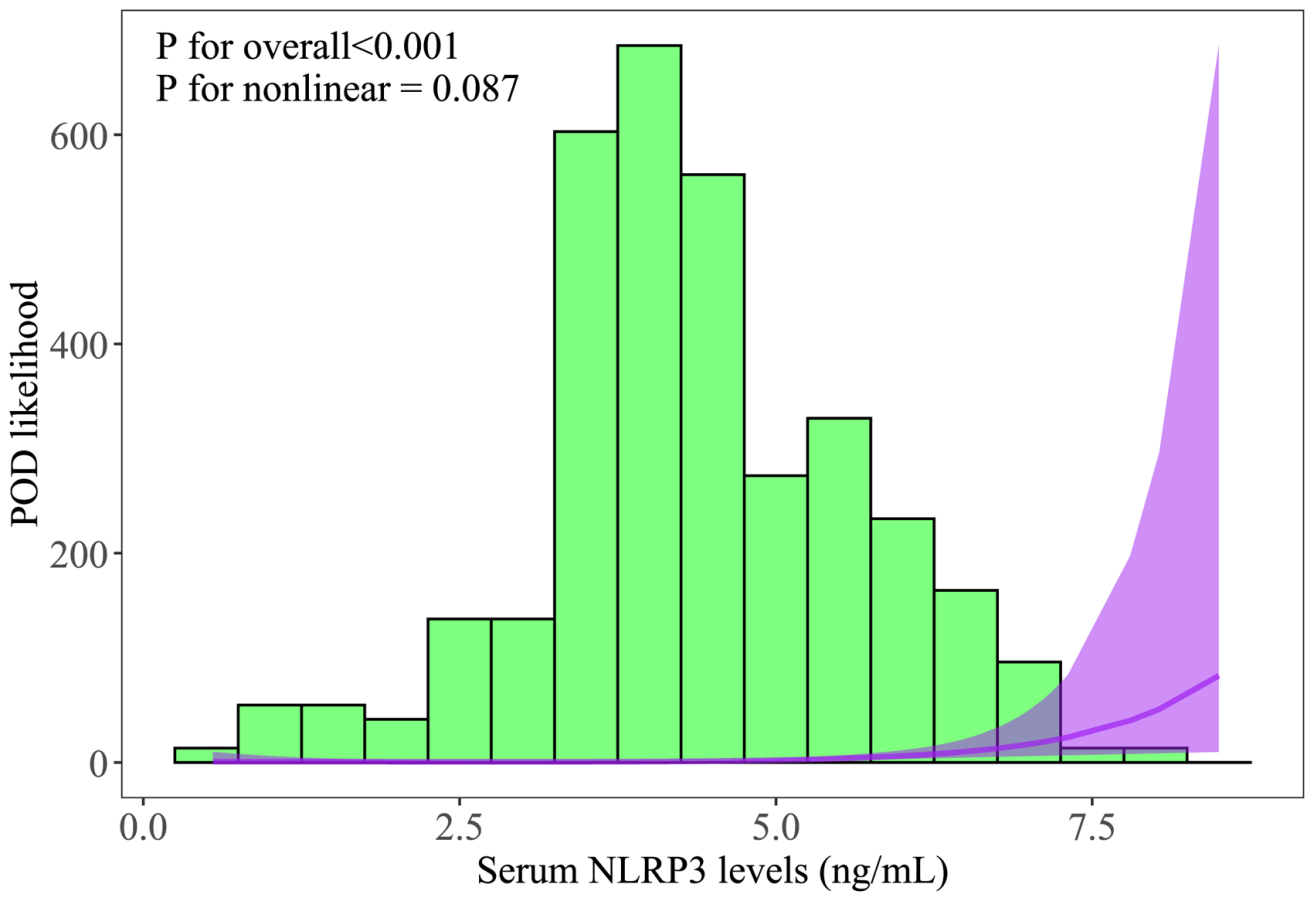
Restricted cubic spline describes the linearity correlation of postoperative serum NLRP3 levels with the probability of postoperative delirium. Postoperative serum NLRP3 levels showed a linear relation to the possibility of postoperative delirium (*p* for non-linear > 0.05). NLRP3 indicates NOD-like receptor protein 3; POD, postoperative delirium.

**Table 2:** Factors in relation to postoperative delirium in elderly patients with lung cancer.

Parameters	Postoperative delirium		*p* value
	Presence	Absence	
Number	53	213	
Age (years)	72 (69–75)	71 (68–74)	0.092
Gender (male/female)	33/20	115/98	0.278
Body mass index (kg/m^2^)	23.0 (20.9–25.4)	23.7 (21.3–25.8)	0.404
Tobacco smoking	17 (32.1%)	43 (20.2%)	0.064
Alcohol drinking	12 (22.6%)	60 (28.2%)	0.418
Hypertension	17 (32.1%)	87 (40.8%)	0.242
Diabetes mellitus	11 (20.8%)	31 (14.6%)	0.268
Hyperlipidemia	15 (28.3%)	61 (28.6%)	0.961
Lung resection method (wedge resection/segmentectomy/lobectomy/pneumonectomy)	14/21/17/1	81/76/50/6	0.366
Surgical time (min)	116 (82–132)	92 (72–125)	0.134
Intraoperative blood loss (mL)	100 (70–140)	80 (60–120)	0.085
Propofol dosage (mg)	1,038 (772–1,146)	750 (656–1,003)	<0.001
Arterial systolic pressure (mmHg)	132 (128–137)	134 (128–139)	0.113
Arterial diastolic pressure (mmHg)	80 (75–82)	79 (73–82)	0.537
American society of anesthesiologists classifications	3 (2–3)	2 (1–2)	<0.001
Eastern cooperative oncology group scores	2 (2–2)	1 (1–2)	<0.001
Charlson comorbidity index scores	4 (3–5)	3 (2–4)	<0.001
Preoperative blood leucocyte counts (×10^9^/L)	7.5 (6.5–8.5)	7.0 (6.1–7.9)	0.070
Preoperative blood C-reation protein levels (mg/L)	7.7 (4.8–11.5)	5.3 (3.7–9.3)	0.011
Preoperative blood glucose levels (mmol/L)	5.4 (5.0–6.5)	5.2 (4.4–6.2)	0.064
Preoperative serum NLRP3 levels (ng/mL)	1.8 (1.4–2.5)	1.6 (1.3–2.1)	0.034
Postoperative blood leucocyte count (×10^9^/L)	9.0 (8.0–10.5)	8.6 (7.6–9.7)	0.077
Postoperative blood C-reation protein levels (mg/L)	14.4 (8.4–17.6)	7.8 (5.8–14.0)	<0.001
Postoperative blood glucose levels (mmol/L)	6.2 (5.3–7.6)	5.9 (4.1–7.2)	0.117
Postoperative serum NLRP3 levels (ng/mL)	5.6 (4.6–6.4)	4.1 (3.5–4.7)	<0.001

Variables were presented as counts (percentages) or medians (percentiles 25th-75th) as applicable. The statistical methods included the chi-square test, Fisher exact test and Mann–Whitney U test. NLRP3 indicates NOD-like receptor protein 3.

**Table 3:** Area under curve of various factors for postoperative delirium of elderly patients.

Parameters	AUC (95% CI)	Threshold	Sensitivity (%)	Specificity (%)	*p* value	*p* value
CCI	0.761 (0.694–0.829)	3	64.15	74.65	0.9607	0.0257
ASA	0.750 (0.679–0.822)	2	56.60	84.98	0.8552	0.0006
Serum NLRP3 levels	0.759 (0.674–0.843)	5.1 ng/mL	69.81	82.16	Reference	0.0032
CCI combined with ASA	0.804 (0.736–0.872)	0.22	64.15	84.51	-	0.1235
Nomogram model	0.842 (0.773–0.910)	0.19	81.13	79.34	-	Reference

CCI indicates Charlson Comorbidity Index; ASA, American Society of Anesthesiologists classifications; NLRP3, NOD-like receptor protein 3; AUC, area under curve; 95% CI, 95% confidence interval.

**Figure 5:**
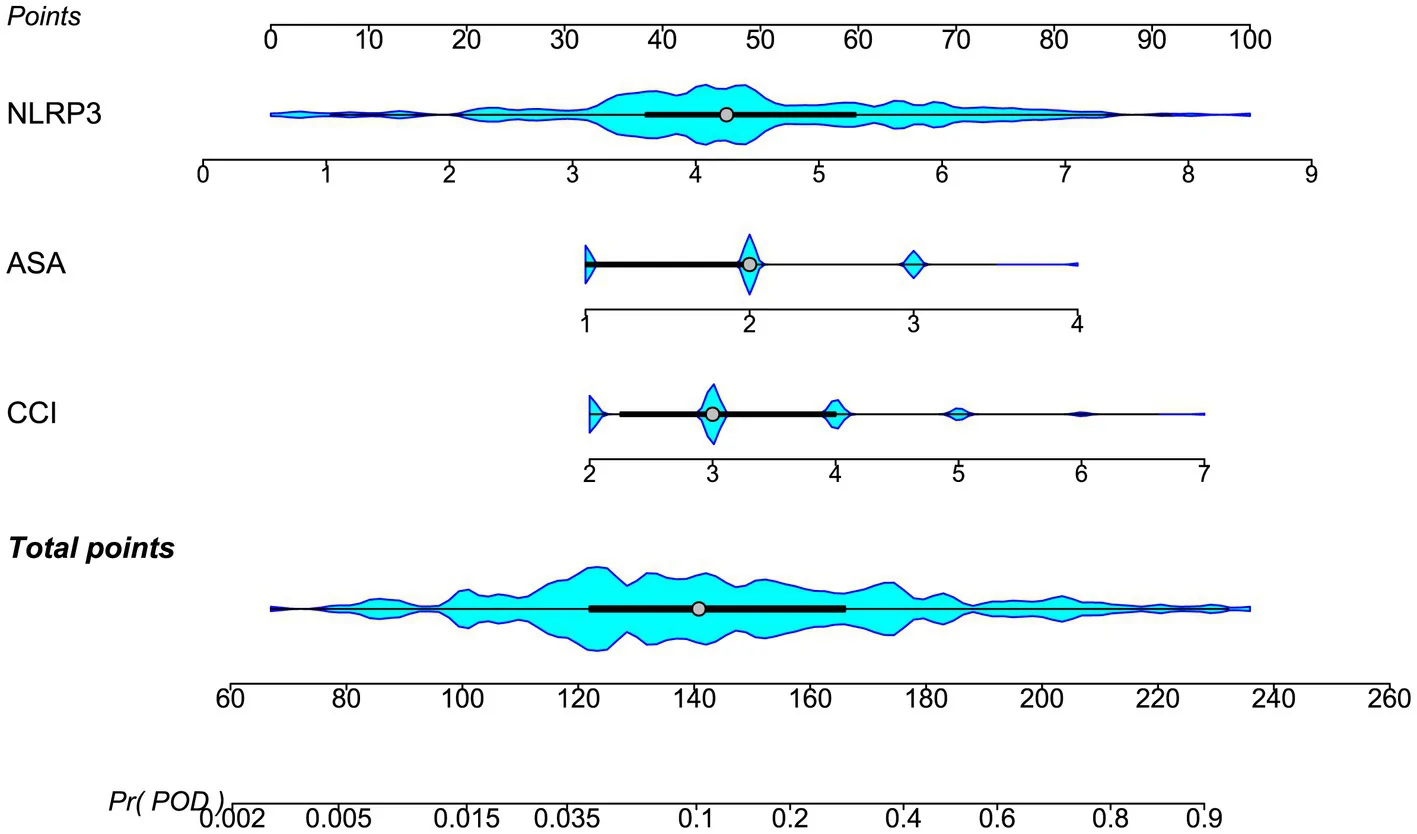
Nomogram model predicts postoperative delirium. The model, including the American Society of Anesthesiologists scale, Charlson comorbidity index scale, and postoperative serum NLRP3 levels, was visualized by the nomogram, so as to anticipate postoperative delirium. ASA means American Society of Anesthesiologists; NLRP3, NOD-like receptor protein 3; CCI, Charlson comorbidity index.

**Figure 6:**
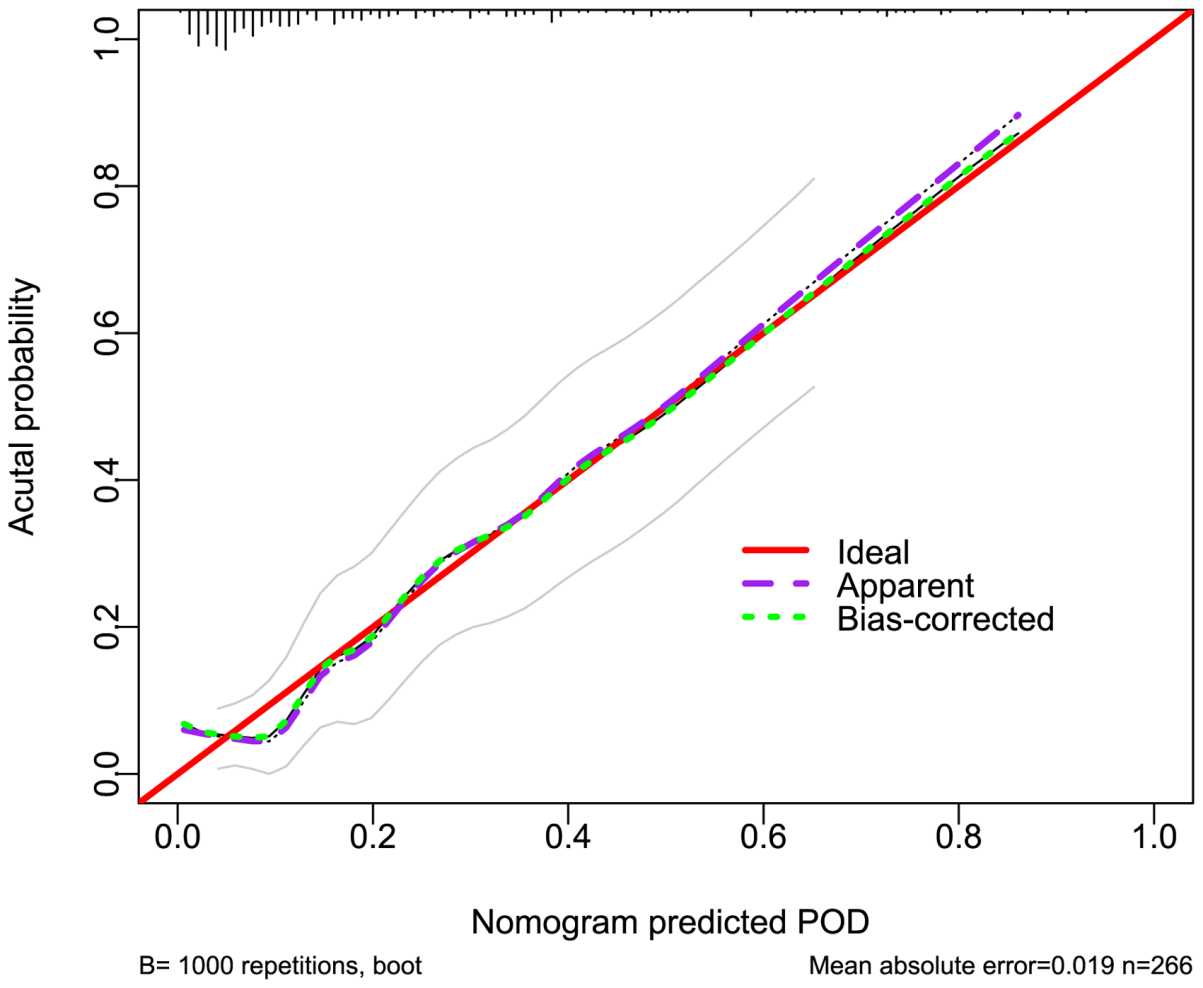
Calibration curve displays stability of the nomogram model for predicting postoperative delirium. The nomogram model kept rather stable in predicting postoperative delirium. POD indicates postoperative delirium.

**Figure 7:**
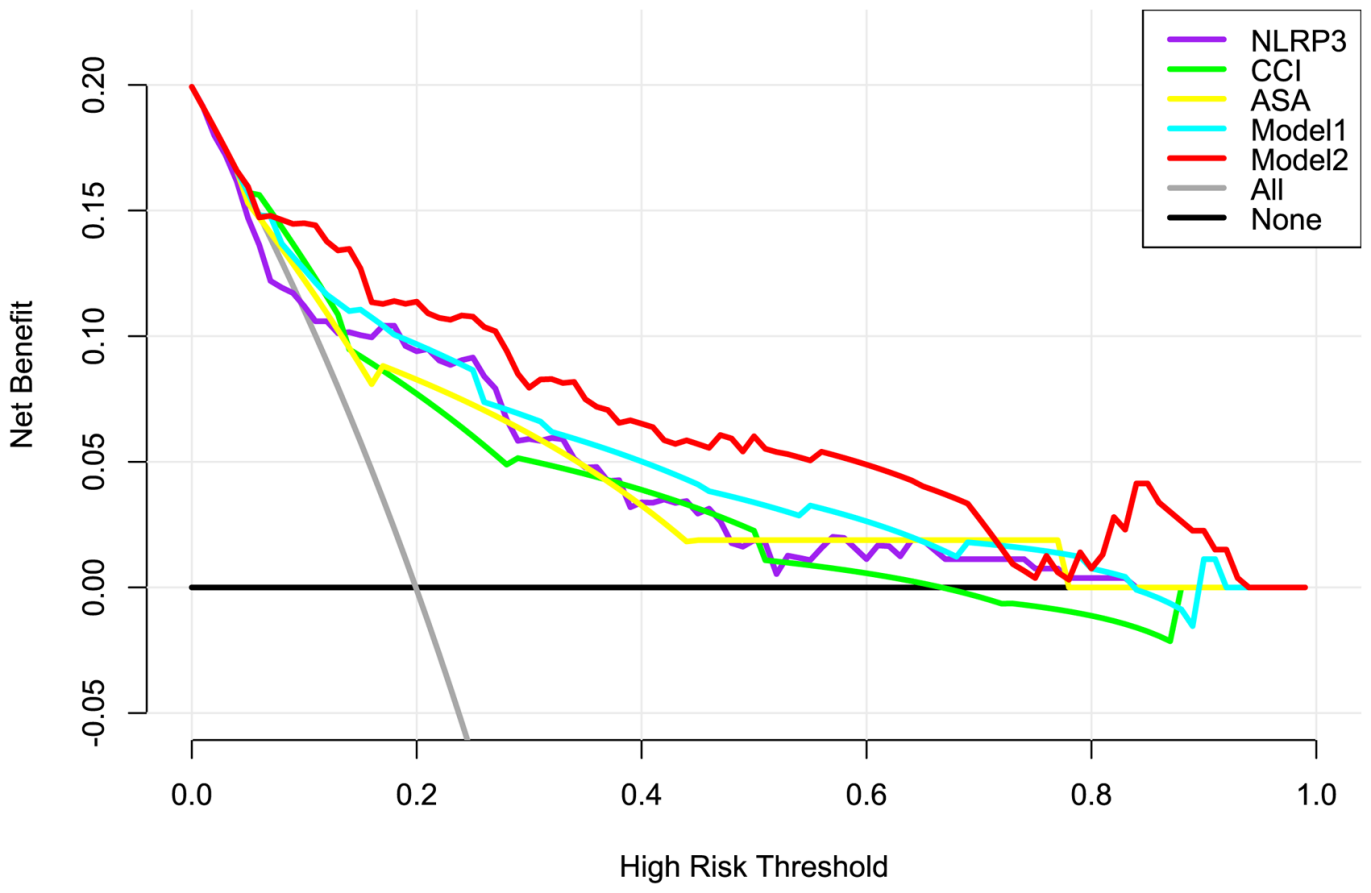
Decision curve demonstrates clinical fit for predicting postoperative delirium. Model 1 included the American Society of Anesthesiologists scale and Charlson comorbidity index scale. Model 2 included the American Society of Anesthesiologists scale, Charlson comorbidity index scale, and postoperative serum NLRP3 levels. ASA means American Society of Anesthesiologists; NLRP3, NOD-like receptor protein 3; CCI, Charlson comorbidity index.

## 4 Discussion

To our knowledge, this is the first investigation to demonstrate the role of serum NLRP3 levels in predicting POD in elderly patients with lung cancer. Our principal findings are that (1) postoperative serum NLRP3 levels were significantly higher than preoperative levels in patients with lung cancer, and serum NLRP3 levels were lowest in controls; (2) postoperative blood CRP levels and ECOG scores were independently correlated with postoperative serum NLRP3 levels; (3) postoperative serum NLRP3 levels, ASA classifications and CCI were independently associated with POD in these patients; (4) the model incorporating the three independent predictors of POD demonstrated good performance across a variety of statistical approaches. Collectively, postoperative serum NLRP3 levels may be strongly associated with POD in elderly patients with lung cancer.

POD, one of the most frequent postoperation complications, is not well recognized in patients with lung cancer, particularly elderly patients. The CAM algorithm is widely accepted for identifying delirium, thus being used for diagnosing POD in this group of elderly patients with lung cancer (27–29). POD incidence varies from 13 to 50% following major non-cardiovascular surgery (30). In this cohort of elderly lung cancer patients undergoing thoracoscopic surgery, its incidence was 19.9% (53/266). As populations age, an increasing proportion of surgical candidates present with multiple comorbidities and limited physiological reserve, which substantially increases vulnerability to POD (5, 26). Rather than a single disease entity, POD is now generally recognized as a complex clinical syndrome arising from the interaction between perioperative stressors and age-related susceptibility of the central nervous system (31, 32). In line with the previous study (33), age and operative procedures were not yet predictors of POD in this group of patients with lung cancer. ECOG is a well-accredited tool for assessing preoperative physical status in oncology, and its extensive prevalence attests to its proven usability in terms of aid in stratification of patient treatment (34). Demonstrably, ECOG was independently associated with POD in elderly patients with gastric cancer (8). In the present study, ECOG was not an independent predictor of POD in elderly individuals with lung cancer. A small sample size of patients with POD may explain this discrepancy. Frail elderly individuals are susceptible to atherosclerosis and endothelial dysfunction, and the two adverse pathological appearances are apt to cause disturbance in noradrenergic/cholinergic neurotransmission, perioperative hypoxia, microembolisms, hypotension, excessive inflammatory response, and elevated oxidative stress, therefore incurring imperceptible brain injury (32, 35–37). Thus, preoperative frailty may predispose patients to physiological insults, thereby increasing the likelihood of POD development. Consistently, ASA classifications and CCI were independent predictors of POD in this cohort of elderly patients with lung cancer. Altogether, these findings suggest that the functional state of cancer patients may be closely relevant to the occurrence of POD.

POD may be attributed to direct injury to brain tissues from perioperative hypoxia, microembolisms, and hypotension; in addition, the propagation of peripheral inflammatory signals to the brain and resultant neuroinflammatory activation appear to play central roles (36, 38–40). Surgical trauma and anesthesia can disrupt blood–brain barrier integrity, allowing circulating inflammatory mediators to enter the central nervous system and activate microglia. This process interferes with neuronal signaling and network stability and is particularly pronounced in older individuals, in whom cognitive reserve and compensatory capacity are already reduced (41–43). CRP is an acute-phase protein with proinflammatory properties. Postoperative blood CRP levels, rather than preoperative levels, have more frequently been associated with POD (44). Taken together, surgical procedures possibly aggravate inflammatory processes, thereby promoting the occurrence of POD.

NLRP3 inflammasome represents a key molecular platform linking peripheral inflammation to central immune activation. Upon sensing danger-associated molecular patterns released during tissue injury or infection, NLRP3 assembles with the adaptor protein ASC and pro-caspase-1, leading to caspase-1 activation and subsequent maturation of interleukin-1β and interleukin-18. This cascade amplifies inflammatory signaling and, under sustained activation, may contribute to neuronal dysfunction and cell injury (45–47). NLRP3 has been increasingly implicated in lung cancer biology. Experimental and clinical studies have suggested that NLRP3 activation influences the tumor immune microenvironment by promoting the recruitment and activation of tumor-infiltrating immune cells (48). Intracellular inflammasome components may be released into the extracellular space during inflammasome activation and inflammatory cell death. Baroja-Mazo et al. showed that activated macrophages can release oligomeric NLRP3 inflammasome particles, which remain biologically active and can be taken up by neighboring cells (49). Similar extracellular NLRP3-containing particles have also been observed following pyroptotic cell death (50, 51). Evidence from human samples further supports circulating NLRP3. Anderson et al. detected NLRP3 in plasma, mainly within CD81-positive extracellular vesicles together with other inflammasome-related proteins, suggesting that circulating NLRP3 may be associated with inflammasome activation and pyroptosis (52). In addition, ASC/NLRP3-positive extracellular inflammasome specks have been identified in the serum of patients with systemic autoinflammatory diseases (50). Previous studies have found that elevated NLRP3 expression has been associated with enhanced inflammatory signaling, tumor cell proliferation, and unfavorable clinical outcomes in lung cancer patients (53). In our study, preoperative serum NLRP3 levels were significantly higher in patients with lung cancer than in controls. Hence, NLRP3 may reflect a broader inflammatory state relevant to tumor progression and subsequent systemic responses.

In this study, postoperative serum NLRP3 levels were substantially higher than preoperative levels in this group of elderly patients with lung cancer undergoing thoracoscopic surgery. In the perioperative setting, the observed increase in serum NLRP3 levels is likely to be driven by multiple factors. Evidently, NLRP3 can be produced by brain tissues in ischemic, hemorrhagic, or traumatic injury; and clinically, blood NLRP3 has been shown to be a biomarker of brain injury (54–56). Moreover, some adverse events, such as hypoxia, hypotension, and microembolisms, may be induced intraoperatively. Thus, serum NLRP3 may be released from the central nervous system. Surgical tissue injury and platelet activation release damage-associated molecular patterns, including adenosine triphosphate, uric acid crystals, high-mobility group box 1, and mitochondrial DNA (45, 57). In parallel, ischemia–reperfusion injury and mitochondrial dysfunction lead to excessive reactive oxygen species production, further promoting NLRP3 inflammasome assembly and activation. The resultant release of pro-inflammatory cytokines may amplify systemic inflammation (58, 59). Collectively, serum NLRP3 may be derived from peripheral cells. Nevertheless, POD represents a severe manifestation of acute postoperative brain dysfunction, arising from the convergence of neuroinflammation, neurotransmitter imbalance, stress-related neuroendocrine changes, and circadian rhythm disruption (60). Blood–brain barrier integrity can be damaged in response to surgical trauma and anesthesia. Inflammatory cytokines generated in the periphery can access the brain through a compromised blood–brain barrier, activate microglia, and exacerbate neuronal injury (61, 62). Furthermore, disturbances in the peripheral immune system can lead to brain dysfunction; peripheral NLRP3 inflammasome may be a pathway in the development of cognitive dysfunction. In summary, whether blood NLRP3 originates from the central or peripheral system this possibility does not negate the potential of serum NLRP3 to serve as a biomarker of POD.

The present study provided clinical evidence linking postoperative serum NLRP3 levels to POD in elderly patients undergoing lung cancer surgery. Moreover, postoperative serum NLRP3 levels were independently correlated with postoperative serum levels of the established inflammatory marker CRP, supporting the close relationship between NLRP3 and inflammation following lung cancer surgery. Additionally, NLRP3 levels were closely associated with ECOG scores. Considering that ECOG can reflect the physical status of cancer patients, poor physical status may facilitate of NLRP3 generation via increased susceptibility to injury. Collectively, these associations may provide a theoretical foundation for the role of serum NLRP3 as a predictor of POD in elderly patients with lung cancer. As expected, the predictive performance of postoperative serum NLRP3 levels was comparable to those of the other two predictive metrics of POD in elderly patients with lung cancer, namely, CCI and ASA classifications. All three independent predictors were incorporated to form the nomogram model, and subsequently, numerous statistical approaches showed that postoperative serum NLRP3 levels may contribute additional predictive value. Alternatively, compared with another POD-prediction model only incorporating CCI and ASA, the nomogram model performed better based on the IDI, NRI, AIC, and BIC; although the addition of serum NLRP3 yielded favorable reclassification metrics, the AUC of the nomogram was not significantly higher than that of the clinical CCI + ASA model alone (*p* = 0.1235). Therefore, our findings should not be interpreted as demonstrating a statistically significant improvement in overall discrimination based on AUC. Internal validation via 10-fold cross-validation showed that the nomogram model had good prediction accuracy, as reflected by the optimism-corrected area under the curve. Taken together, postoperative serum NLRP3 may serve as a potential research biomarker warranting further investigation at a preoperative time point and in external cohorts as a marker of inflammatory burden and susceptibility to POD.

Several limitations warrant clarification. First, although our prespecified sample-size estimation indicated that the study had a sufficient number of patients, the generalizability of our conclusions requires further external validation in larger samples. Second, postoperative blood samples were collected in the early morning of postoperative day 1 before the scheduled CAM assessment. However, because a formal CAM assessment was not performed immediately before blood sampling, we could not confirm that all patients were free of delirium at the exact time of sampling. Therefore, reverse causation cannot be excluded; elevated postoperative serum NLRP3 may, at least in some patients, reflect inflammatory changes accompanying already developing delirium rather than preceding its onset. For the same reason, a sensitivity analysis restricted to patients confirmed to be delirium-free at the time of sampling could not be reliably performed using the available data. Future prospective studies should incorporate a standardized delirium assessment immediately before biomarker sampling and subsequently follow delirium-free patients for incident POD initially. Accordingly, the present findings should primarily be interpreted as evidence of an association between postoperative serum NLRP3 levels and POD rather than as definitive evidence of temporal prediction. Third, controls have been investigated for their age, gender, body mass index, tobacco smoking, alcohol drinking, hypertension, diabetes mellitus, and hyperlipidemia. No significant differences were observed between patients and controls with respect to the above eight factors. Admittedly, other confounding factors may affect serum NLRP3 levels, but were not investigated currently. This comparison of serum NLRP3 levels between patients and controls may be considered exploratory only. Fourth, twice-daily assessment over only 3 days may miss hypoactive, nocturnal, and late-onset delirium (which can occur after day 3), potentially underestimating the incidence. Moreover, POD assessments were currently done by a physician trained in CAM administration who was blinded to the serum NLRP3 results, so no actual inter-rater reliability was reported. In the future, it is better to lengthen assessment duration and add nocturnal appraisal to precisely identify POD in patients, and this study can be completed by at least two physicians. Finally, some confounding factors, such as anesthesia technique, analgesia/sedation strategy, opioid consumption, and postoperative sleep quality, were not collected because they can be very difficult to obtain quantitative assessments. Nevertheless, a variable selection strategy has been implemented rigorously in order to assure robustness of regression results, but the influence of those unmeasured perioperative factors (such as analgesia/sedation strategies, postoperative sleep quality, and subclinical infection states) on the findings cannot be entirely ruled out; so future studies should be more meticulously designed.

## 5 Conclusion

Significantly elevated postoperative serum NLRP3 levels, which were closely associated with ECOG scores and postoperative blood CRP levels, are independently associated with POD in elderly patients with lung cancer. Furthermore, postoperative serum NLRP3 levels and the combined model demonstrate satisfactory predictive value for POD. Nonetheless, this study only provides hypothesis-generating evidence, and future studies will be directed toward evaluation of the marker at a preoperative time point, performing external independent validation, and comparing its cost-effectiveness with that of routine metrics.

## Data Availability

The raw data supporting the conclusions of this article will be made available by the authors, without undue reservation.
