# Supplementary material for: Serum NLRP3 levels are associated with postoperative delirium of elderly patients with lung cancer: an observational analytical study
Source: Front Neurol. 2026 Sep 29;17:1868771. doi: 10.3389/fneur.2026.1868771 (PMC13658091; doi:10.3389/fneur.2026.1868771)
Supplement: Supplementary file 1 [file Supplementary_file_1.docx]

**Figure legends**

**Supplementary Figure 1**

Correlogram displaying correlation between postoperative serum NLRP3 levels and ECOG scores.

Postoperative serum NLRP3 levels were evidently related to ECOG scores (p < 0.001).

NLRP3 stands for NOD-like receptor protein 3; ECOG, Eastern Cooperative Oncology Group.

**Supplementary Figure 2**

Violin plot delineating postoperative serum NLRP3 levels across ECOG scale.

Postoperative serum NLRP3 levels were apparently lowest in subgroup with ECOG score 0, followed by those with the scores 1 and 2, and were obviously highest in subgroup with the score 3 (^*^ p < 0.05; ^**^ p < 0.01; ^***^ p < 0.001)

NLRP3 signifies NOD-like receptor protein 3; ECOG, Eastern Cooperative Oncology Group.

**Supplementary Figure 3**

Correlogram exhibiting relevance of postoperative serum NLRP3 levels to blood CRP levels.

Postoperative serum NLRP3 levels were profoundly correlated with postoperative blood CRP levels (p < 0.001).

NLRP3 means NOD-like receptor protein 3; CRP, C-reactive protein.

**Supplementary Figure 4**

Restricted cubic spline depicting linear correlation of postoperative serum NLRP3 levels with ECOG.

There was a linear correlation between postoperative serum NLRP3 levels and ECOG scores (p for nonlinear > 0.05).

NLRP3 denotes NOD-like receptor protein 3; ECOG, Eastern Cooperative Oncology Group.

**Supplementary Figure 5**

Restricted cubic spline outlining linearity connection between postoperative serum NLRP3 levels and postoperative blood CRP levels.

Postoperative serum NLRP3 levels were in a dose-effect manner correlated with postoperative blood CRP levels (p for nonlinear > 0.05).

NLRP3 indicates NOD-like receptor protein 3; CRP, C-reactive protein.

**Supplementary Figure 6**

The least absolute shrinkage and selection operator coefficient profiles for predictor selection.

Least absolute shrinkage and selection operator coefficient profiles of the six candidate variables. Coefficient trajectories are plotted against the logarithm of the penalty parameter (−log λ). As the penalty parameter decreased, the coefficients of candidate variables gradually entered the model. The six candidate variables identified in the univariate analysis were included in the LASSO regression, and ECOG score and postoperative serum C-reactive protein levels were ultimately retained for subsequent multifactorial linear regression analysis.

ECOG indicates Eastern Cooperative Oncology Group.

**Supplementary Figure 7**

Selection of the optimal tuning parameter (λ) by 10-fold cross-validation in least absolute shrinkage and selection operator regression.

The optimal penalty parameter (λ) was determined by 10-fold cross-validation. The red dots represent the mean cross-validated mean squared error, and the gray error bars indicate ±1 standard error. The two vertical dashed lines denote the optimal λ values according to the minimum criteria (λ_min) and the one-standard-error criterion (λ_1se), respectively. Based on the optimal λ value, the LASSO regression selected two variables, namely ECOG score and postoperative serum C-reactive protein levels, for inclusion in the subsequent multifactorial linear regression model.

ECOG indicates Eastern Cooperative Oncology Group.

**Supplementary Figure 8**

Violin portraying postoperative serum NLRP3 levels between patients with and without POD.

Postoperative serum NLRP3 levels were substantially higher in patients with POD than in those without this event (^***^ p < 0.001).

NLRP3 indicates NOD-like receptor protein 3; POD, postoperative delirium.

**Supplementary Figure 9**

Least absolute shrinkage and selection operator coefficient profiles of the eight candidate variables.

Least absolute shrinkage and selection operator coefficient profiles of the eight candidate variables. Coefficient trajectories are plotted against the logarithm of the penalty parameter (−log λ). As the penalty parameter decreased, the coefficients of candidate variables gradually entered the model. The eight candidate variables identified in the univariate analysis were included in the Least absolute shrinkage and selection operator regression, and American Society of Anesthesiologists classification, Charlson Comorbidity Index, and postoperative serum NOD-like receptor protein 3 levelS were ultimately retained for subsequent binary logistic regression analysis.

**Supplementary Figure 10**

Selection of the optimal tuning parameter (λ) using 10-fold cross-validation in the least absolute shrinkage and selection operator model.

The optimal penalty parameter (λ) was determined by 10-fold cross-validation. The red dots represent the mean cross-validated mean squared error, and the gray error bars indicate ±1 standard error. The two vertical dashed lines denote the optimal λ values according to the minimum criterion (λ_min) and the one-standard-error criterion (λ_1se), respectively. Based on the optimal λ value, the least absolute shrinkage and selection operator regression selected three variables, namely American Society of Anesthesiologists classification, Charlson Comorbidity Index, and postoperative serum NOD-like receptor protein 3 level, for inclusion in the subsequent binary logistic regression model.

**Supplementary Figure 11**

E value for sensitivity analysis.

E value was calculated for reflecting stability of regression results and its value was 2.7488.

95% CI signifies 95% confidence interval.

**Supplementary Figure 12**

Receiver operating characteristic curve appraising predictive ability of various metrics for postoperative delirium.

The model including ECOG scores, postoperative serum NLRP3 levels and postoperative C-reactive protein levels had significantly highest predictive capability for postoperative delirium (^*^ p < 0.05; ^**^ p < 0.01).

AUC indicates area under curve; 95%	CI, 95% confidence interval; ECOG, Eastern Cooperative Oncology Group; CRP, C-reactive protein; NLRP3, NOD-like receptor protein 3; ns, non-significant.

**Supplementary Figure 13**

Assessment about model improvement rate of nomogram model for predicting postoperative delirium.

New model referred to the nomogram model and standard model was composed of Eastern Cooperative Oncology Group scores and postoperative blood C-reactive protein levels. New model had significantly elevated model improvement rate.

**Supplementary Figure 14**

Internal validation of the nomogram model using 10-fold cross-validation.

Receiver operating characteristic curves from 10-fold cross-validation were used to assess the discrimination performance of the nomogram model. Gray lines represent the receiver operating characteristic curves for individual validation folds, whereas the red line represents the aggregated overall receiver operating characteristic curve. The optimism-corrected area under the receiver operating characteristic curve was 0.825 (95% confidence interval, 0.752–0.899), indicating good discriminative ability of the model.

ROC indicates receiver operating characteristic.

Supplementary Figure 1

Supplementary Figure 2

Supplementary Figure 3

Supplementary Figure 4

Supplementary Figure 5

Supplementary Figure 6

Supplementary Figure 7

Supplementary Figure 8

Supplementary Figure 9

Supplementary Figure 10

Supplementary Figure 11

Supplementary Figure 12

Supplementary Figure 13

Supplementary Figure 14
